# Protein A Detection Based on Quantum Dots-Antibody Bioprobe Using Fluorescence Coupled Capillary Electrophoresis

**DOI:** 10.3390/ijms15021804

**Published:** 2014-01-24

**Authors:** Lin Qiu, Yanhua Bi, Cheli Wang, Jingyan Li, Peilin Guo, Jinchen Li, Weijiang He, Jianhao Wang, Pengju Jiang

**Affiliations:** 1 State Key Laboratory of Coordination Chemistry, Coordination Chemistry Institute, School of Chemistry and Chemical Engineering, Nanjing University, Nanjing 210093, China; E-Mails: linqiupjj@gmail.com (L.Q.); heweij69@nju.edu.cn (W.H.); 2 School of Pharmaceutical Engineering and Life Science, Changzhou University, Changzhou 213164, China; E-Mails: clwang@cczu.edu.cn (C.W.); jingyan.leee@gmail.com (J.L.); 351533126@163.com (P.G.); 15895072832@163.com (J.L.); 3 Shandong Academy of Medical Sciences, Jinan 250062, China; E-Mail: byh168@163.com

**Keywords:** QDs, FRET, bioprobe, capillary electrophoresis, protein A

## Abstract

In this report, fluorescence detection coupled capillary electrophoresis (CE-FL) was used to detect Protein A. Antibody was first labeled with Cy5 and then mixed with quantum dots (QDs) to form QDs-antibody bioprobe. Further, we observed fluorescence resonance energy transfer (FRET) from QDs donor to Cy5 acceptor. The bioprobe was formed and brought QDs and Cy5 close enough to allow FRET to occur. After adding protein A, the FRET system was broken and caused the FRET signal to decrease. Thus, a new method for the determination of protein A was proposed based on the FRET signal changes. This study provides a new trail of thought for the detection of protein.

## 1. Introduction

Compared with traditional organic dyes, the QDs developed in recent years have many attractive features, including high photobleaching threshold, good chemical stability, relatively narrow and symmetric luminescence bands [1]. QDs’ unique optical properties make it an ideal choice for luminescent biological probes [1–4].

Many strategies have now been developed to conjugate QDs with biomolecules for the preparation of QDs based bioprobes, for example: covalent conjugation method [5], metal-affinity driven self-assembly method [6,7] and electrostatic adsorption method [8,9]. Among them electrostatic adsorption method is the most simple and direct method. The interaction between QDs and biomolecules was not stable enough and it can be easily displaced by other molecules. Therefore, the bioprobes prepared in this method can be used to detect biomolecules.

QD-based FRET biosensors have been widely used in immunoassay [10], biomedical sensor [11,12] and intermolecular binding assay [13,14]. Capillary electrophoresis has numerous applications and provides the advantage of high resolution, speed, ease of use, automation and low cost. Combine CE and FRET technology, we have studied the interaction of protein and nanoparticles [15], the protein peptide interaction [16] and enzyme detection [17], *etc.* Recently, Chang *et al.*, reported QDs-based CE-FL for the ultrasensitive detection and quantification of acidic disaccharides [18].

In this report, we prepared QDs-IgG-Cy5 bioprobe by electrostatic adsorption method. The results indicated that one QD could absorb four IgG molecules. FRET happened between QDs and IgG-Cy5. With adding protein A, the FRET signals decrease gradually and this method can be used for protein A detection.

## 2. Results and Discussion

Many methods are widely applied to study biomolecule interaction, such as surface plasmon resonance (SPR) [19], enzyme-linked immunosorbent assay (ELISA) [20], high perfomance size exclusion chromatography (HPSEC) [21], capillary electrophoresis (CE) [16] and others. Especially in recent, Zhao *et al.*, reported a simple but efficient electrochemical method to probe into the interaction between β-amyloid peptides and bilayer lipid membrane for revealing the toxic mechanism of Alzheimer’s disease [22]. This method might provide a convenient and powerful approach for *in vitro* studies of diseases.

CE-FL has been shown to be an effective method to detect QDs-protein interaction, which reveals subtle changes in the structure and composition of the surface bound ligands on QDs [15,23]. Furthermore, CE-FL can provide far more detailed information on QDs-protein assembly than ensemble fluorescence measurement [15].

**Figure 1.**
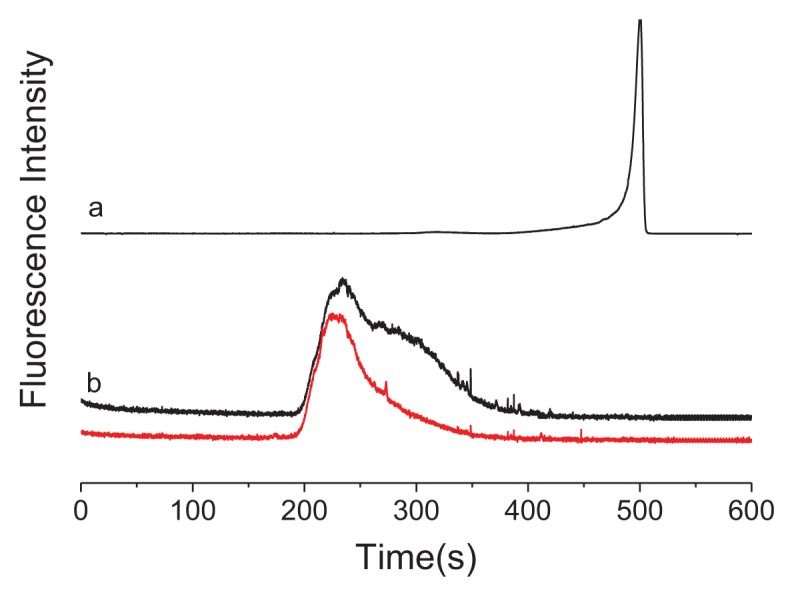
Electropherograms of quantum dots (QDs)-IgG-Cy5 conjugation with detection in 612 nm channel (Black) and 670 nm channel (Red). (**a**) QDs alone, 2 μM and (**b**) QDs-IgG-Cy5, 2 μM. CE conditions: 25 mM borate buffer (pH 9.3) at 18 kV. λ_ex_ = 420 nm.

In our CE experiments, two signal channels of fiber optic spectrometer with fixed detecting wavelength at 612 ± 10 and 670 ± 10 nm were used to simultaneously collect the fluorescence signal of donor and acceptor. QDs and IgG-Cy5 mixtures were first analysed by CE-FL. QDs-IgG-Cy5 bioprobe was prepared by electrostatic adsorption method. When mixed with QDs in solution, IgG-Cy5 can assemble with QDs. Figure 1 shows the electropherograms of mixing IgG-Cy5 with QDs. It was obvious that CE could efficiently separate the bound and unbound species. The migration time of QDs alone was 500 s (Figure 1, curve a); while for the conjugates (Figure 1, curve b), indicated by a stable species of QDs-IgG-Cy5 in CE-FL with migration time of 230 s, significantly different from QDs. By the location of the emission peak, this peak was known to be caused by the QDs-IgG-Cy5. After the conjugation of IgG-Cy5 and QDs, the surface charge changed and the fluorescence peak moved forward. This implies an ordered assembly. Experimental results also approved that there was cross-talk between the donor and acceptor channel. This indicated that FRET happened between QDs and Cy5.

**Figure 2.**
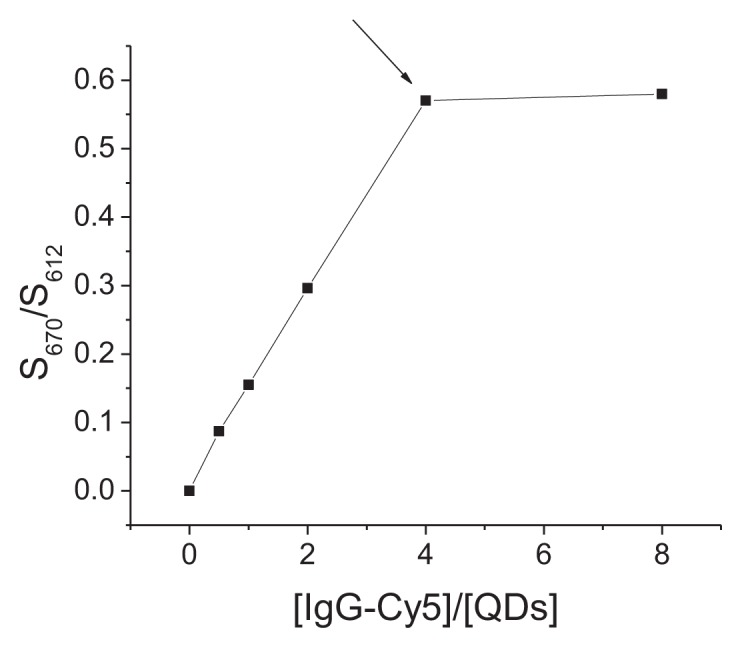
*S*_670_/*S*_612_ increases with increasing [IgG-Cy5]/[QDs] ratio. *S*_670_ and *S*_612_ are integrated signal intensity at 670 nm (acceptor channel) and 612 nm (donor channel), respectively. CE conditions: 25 mM borate buffer (pH 9.3) at 18 kV. λ_ex_ = 420 nm. [QDs] = 2 μM.

In order to choose the optimal ratio of QDs to IgG-Cy5, the conjugation of QDs with different concentration of IgG-Cy5 was detected by CE-FL. With increasing the ratio of IgG-Cy5/QD, the FRET signals increased gradually. FRET signals reached a plateau when the ratio excessed 4 (Figure 2). This indicated that one QD could bind approximately four IgG molecules.

**Scheme 1.**
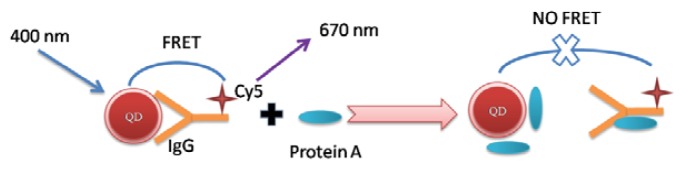
Schematic illustration of the fluorescence resonance energy transfer (FRET) between QDs and Cy5.

Protein A is a surface protein found in the cell wall of the bacteria *Staphylococcus aureus*. This protein has an ability to bind immunoglobulins through interaction with their Fc region [24]. Therefore, if protein A exists in the QDs-IgG-Cy5 solution, it would break the FRET system and form QDs-protein A and protein A-IgG-Cy5. This will result in the change of the FRET signals (Scheme 1). Based on this, The CE-FL method was used to investigate the dynamic displacement of surface bound IgG-Cy5 by protein A.

**Figure 3.**
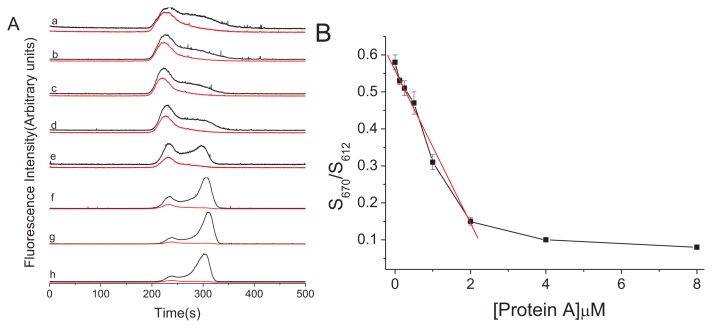
(**A**) Electropherograms of displacement of QD bound IgG-Cy5 by different amount of protein A (black: 612 nm for QDs; red: 670 nm for Cy5). a, 0 μM; b, 0.125 μM; c, 0.25 μM; d, 0.5 μM; e, 1 μM; f, 2 μM; g, 4 μM; h, 8 μM; and (**B**) Protein A detection by the changes of *S*_670_/*S*_612_ [QDs-IgG-Cy5] = 2 μM.

We examined the changes in the properties of the QDs in response to protein A concentration using CE-FL. Increasing amounts of protein A were mixed with a constant concentration of QDs-IgG-Cy5 for 10 min. It was found that QDs showed a low emission intensity in the protein A concentration range of 0–1.0 μM. The QDs-IgG-Cy5 bioprobe was formed and the mutual affinity of the QDs and IgG brought QDs and Cy5 close enough to allow FRET to occur, which resulted in the decrease of the emission intensity of QDs and increase intensity of Cy5. Besides, incubation of 4 μM of protein A almost completely displaced the surface bound IgG-Cy5 (Figure 3A, curve g). A new peak in 320 s was appeared with fluorescence signal only at 612 nm, which might be caused by QDs-protein A (Figure 3A, curves e–h). At the same time, the peak of QDs-IgG-Cy5 at 230 s decreased gradually with the increment of protein A concentration.

Furthermore, it was found that the change of the FRET signals from QDs to Cy5 was obvious with the increase of protein A. The results showed that the FRET signals decreased linearly with the concentration of protein A in the range of 0–2 μM (Figure 3B).

## 3. Experimental Section

### 3.1. Materials and Instruments

Protein A and Human IgG were purchased from BeiJing Cowin Biotech Co. Ltd. (Cowin Biotech, Beijing, China). Glutathione (GSH) was purchased from Adamas-Beta Co. Ltd. (Adamas-Beta, Shanghai, China). All other chemicals and materials were of analytical grade. Ultrapure water (≥18.2 mΩ) purified by Milli-Q system (Millipore, Bedford, MA, USA) was used for preparation of all solutions. The electrophoresis buffers were filtered through a 0.22 μm filter before use.

Capillary electrophoresis analyses with fluorescence detection were carried out on a home-built system, consisting of a high voltage supply (0–30 kV) (Shanghai Nuclear Research Institute, Shanghai, China), a fused-silica capillary with an inner diameter (ID) of 75 μm (Yongnian Optical Fibre Factory, Hebei, China) and an inverted IX71 fluorescence microscope (Olympus, Tokyo, Japan) equipped with a 100 W mercury lamp, an excitation filter (BP 420 ± 20 nm), a dichromatic mirror (DM 455) and a fiber optic spectrometer QE65000 (Ocean Optics, Dunedin, FL, USA) attached to the side port.

### 3.2. Preparation of GSH Stabilized QDs

Briefly, oil-soluble CdSe–ZnS core-shell QDs were purchased from JIAYUAN Quantum Dots Co. Ltd. (JIAYUAN, Wuhan, China) and dissolved in chloroform to 8.0 μM. GSH stabilized QDs were synthesized based on the previously reported procedures of the exchange of TOPO on the surface of as-synthesized QDs by GSH [25]. QDs were dissolved in chloroform, to which a 100 μL GSH solution (containing 0.142 g GSH and 40 mg KOH in 2 mL methanol) was added followed by vigorous shaking. After the addition of 1.5 mL NaOH aqueous solution (1 mM), the top aqueous layer was separated and precipitated with NaCl and methanol to remove excess GSH. The resulting QDs were dissolved in 500 μL borate buffer (pH 8.5, 10 mM). The concentration of QDs was measured based on the previously reported method [26].

### 3.3. Preparation of Cy5-NHS

Cy5 free acid was synthesized based on the previously reported procedures [27]. Dissolve Cy5 free acid and TSTU at a ratio of 1:1 in DMF, add 2 fold of DIPEA and then vortex for 1 h. DMF was then removed by Eppendorf concentrator plus (Eppendorf, Hamburg, Germany). Preparative HPLC was then used to get purified Cy5-NHS. The identity of Cy5-NHS was confirmed by LC-MS: *m*/*z* calcd for [M + H]^+^ 753.8, found 753.5.

### 3.4. Preparation of IgG-Cy5

A total of 0.5 mg Human IgG was prepared in 0.5 mL PBS buffer (pH 7.2). IgG was labeled by 10 fold of Cy5-NHS at room temperature for 2 h. Excess Cy5-NHS was cleaned up by Zeba-spin desalting column (Pierce, catalog number 89882, Rockford, IL, USA).

### 3.5. Preparation of QDs-IgG-Cy5 Conjugates

Activate the QDs by mixing 10 μL QDs (8 μM) and different concentration of IgG-Cy5 in 30 μL 0.1 M borate buffer. Incubate for 10 min at room temperature with continuous gentle mixing. Precipitation was removed by centrifugation and excess QDs were removed by ultrafiltration. Then different concentration of protein A was added and incubated for 10 min at room temperature. The purified QDs-IgG-Cy5 was dispersed in borate buffer (pH 7.4). It was stable at 4 ºC for one week.

### 3.6. Procedure of Capillary Electrophoresis

CE experiments were all performed in 75 μm ID × 60 cm long fused-silica capillaries. The effective length (length from injection to the detection window) was 35 cm. When a capillary was firstly used, it was rinsed with 0.1 M HCl, pure water, 0.1 M NaOH, pure water and electrophoretic buffer sequentially for 20 min, respectively. Hydrodynamic injection was performed by siphoning at 15 cm height differences for 20 s at anode. A solution of 25 mM Na_2_B_4_O_7_ (pH 9.3) was used as CE separation buffer. Before analysis, the capillary was injected by high pressure and equilibrated with running buffer for 15 min. The separation was achieved at room temperature. Between each runs, the capillary was washed with running buffer for 10 min to ensure the reproducibility.

## 4. Conclusions

In conclusion, we have systematically studied QDs and IgG-Cy5 interaction using CE-FL. CE-FL provides a facile, fast, highly sensitive, relatively inexpensive and disposable device for rapid measurement of ligand-particle interaction. A new method for the determination of protein A was established based on the FRET signal changes. Under the optimal conditions, this method showed good sensitivity. This method can be used to measure protein and may widen the application of QDs.
